# Analyzing the potential targets and mechanisms of liver damage induced by acetyl tributyl citrate plasticizer using network toxicology, molecular docking and *in vitro* experiments

**DOI:** 10.3389/fphar.2025.1636576

**Published:** 2025-07-16

**Authors:** Dong-qun Guo, Yu-ming Fang, Zhen-dong Sun, Ya-fen Zeng, Gui-dan Wang, Jin-wei Liang

**Affiliations:** ^1^ Department of Urology, The Second Affiliated Hospital of Fujian Medical University, Quanzhou, China; ^2^ Department of Anesthesiology, The Second Affiliated Hospital of Fujian Medical University, Quanzhou, China

**Keywords:** network toxicology, molecular docking, ATBC, liver damage, vitro experiments

## Abstract

**Background:**

Acetyl tributyl citrate (ATBC) may have adverse effects on liver health; however, the underlying mechanisms and pathophysiology remain unclear. The objective of this study was to elucidate the complex effects of ATBC on the liver and to determine the underlying molecular mechanisms by which environmental pollutants affect the disease process.

**Methods:**

We used network toxicology and molecular docking techniques to analyze potential targets and mechanisms of liver injury caused by ATBC plasticizer. Potential targets associated with ATBC exposure and liver injury were identified by using ChEMBL, STITCH, GeneCards and OMIM databases. Enrichment analysis was performed using the DAVID database (https://david.ncifcrf.gov/) to identify biological pathways associated with these genes. Finally, transcription quantitative polymerase chain reaction, CCK-8 assay, Western blot, and immunofluorescence staining were used to assess the effect of candidate potential targets on liver injury.

**Results:**

A total of 74 common targets associated with ATBC and liver injury were obtained. Enrichment analysis emphasized the association between these plastocyanin-targeted genes and the apoptotic pathway, suggesting that plastocyanin has a broad impact on cell survival. Moreover, molecular docking analysis demonstrated that ATBC exhibited a specific binding affinity for TNF-α, thereby suggesting that TNF-α plays a pivotal role in the regulation of liver damage pathogenesis. *In vitro* experiments further validated the expression of this molecule with the apoptosis marker molecules BAX and Bcl2 in ATBC-induced liver injury.

**Conclusion:**

The study suggests that TNF-α is involved in the process of ATBC-induced liver damage and may be related to cell apoptosis.

## 1 Introduction

Acetyl tributyl citrate (ATBC), a significant citrate ester, is widely used in industrial and daily applications. It is synthesized by the esterification of citric acid and n-butanol in the presence of a catalyst (Zhang et al., 2023; Kim et al., 2018). ATBC serves as an environmentally friendly plasticizer for materials such as polyvinyl chloride (PVC), thanks to its advantageous plasticizing characteristics, low toxicity, and biocompatibility (Kim et al., 2018; Ren et al., 2022). Compared to conventional phthalate plasticizers, ATBC boasts several advantages, including low volatility and excellent water resistance. As a result, it is deemed an ideal alternative to phthalate esters (e.g., DEHP) (Fan et al., 2020) and finds application in various products, such as medical devices, food packaging, children’s toys, cosmetics, and personal care items (Gong et al., 2021).

Nevertheless, the increasing utilization of ATBC has heightened attention toward its potential impacts on human health. Although it is generally recognized as a low-toxicity substance, emerging research suggests that long-term or high-dose exposure to ATBC could have adverse effects on living organisms (Alves et al., 2017; Testai et al., 2016). The liver, a vital organ responsible for maintaining normal physiological functions (Jenne and Kubes, 2013), is particularly affected by exposure to exogenous chemicals. Studies have shown that plasticizers can induce oxidative stress and disrupt the liver’s metabolic enzyme systems, thereby affecting lipid metabolism upon entering the human body (Íñigo-Catalina et al., 2024; Lee et al., 2020; Kumar et al., 2024). Given the widespread presence of ATBC in both the environment and the human body, it is crucial to investigate its relationship with liver damage.

Traditional toxicological approaches primarily focus on direct toxic effects of individual chemicals on the liver. However, this methodology is inadequate for elucidating the toxicity mechanisms of ATBC and its metabolites within complex biological systems. Network toxicology, an emerging interdisciplinary field, integrates principles from systems biology, bioinformatics, and toxicology to explore the interactions between chemical compounds and biomolecules from a holistic network perspective (Tao et al., 2013). This innovative framework provides a new methodology for examining the association between ATBC and liver damage. Network toxicology methodologies facilitate comprehensive analysis of the impacts of ATBC and its metabolites on liver-related biomolecular networks, elucidating potential mechanisms of hepatotoxicity. This scientific foundation can inform evaluations of ATBC’s safety and guide strategies for preventing and treating liver damage associated with its exposure.

The present study aims to delineate the toxicological properties of ATBC and predict its potential toxicity and molecular mechanisms through cytotoxicity assessments. Additionally, this research explores the effects of ATBC exposure on mouse liver cells using cellular models to provide foundational insights for diagnosing liver diseases linked to plasticizers like ATBC.

## 2 Materials and methods

### 2.1 Materials

#### 2.1.1 Reagent

Acetyl tributyl citrate (ATBC, CAS No. 77-90-7, purity >98.5%) was purchased from Sigma-Aldrich (St. Louis, MO), while dimethyl sulfoxide (DMSO) was obtained from Sorabi (Beijing, China). Prior to utilization, ATBC was dissolved in DMSO to formulate a stock solution, which was subsequently diluted with cell culture medium to the desired working concentration.

#### 2.1.2 Cells

Human liver cell line THLE-2 was obtained from Anweisci (Shanghai, China). Cells were cultured in BEGMkit medium (Lonza-Clonetics, CC-3170) in a humidified incubator at 37°C and 5% CO_2_.

### 2.2 Methods

#### 2.2.1 Preliminary network analysis of ATBC toxicity

The use of network algorithms and biotoxicity prediction methods allowed us to predict the toxicity of ATBC compounds using structural models. By employing two software tools altogether, namely, ADMETlab 2.0 and ProTox3 platforms, as tentative screening tools, we aimed to initially evaluate its potential association with liver injury.

#### 2.2.2 Collection of ATBC targets

To comprehensively identify molecular targets associated with ATBC, the standard structure and smile node of ATBC were first searched in the PubChem database (http://pubchem.ncbi.nlm.nih.gov/). Based on this search result, we retrieved the potential targets of ATBC from ChEMBL database, and utilized the STITCH database (https://stitch.embl.de/) to “acetyl tributyl citrate” as the keyword was searched to obtain information about the targets of action of ATBC. The obtained targets were screened and organized to remove duplicates and non-human gene targets, thereby obtaining the action target set of ATBC.

#### 2.2.3 Selection of a network of targets related to liver damage

Utilizing the OMIM database (https://www.omim.org/) and the GeneCards database (https://www.genecards.org/), a search was performed with the keywords “liver damage” as keyword to collect gene targets related to liver damage. The retrieved targets were summarized and de-emphasized to obtain the set of liver damage-related targets.

#### 2.2.4 Construction of protein interaction networks and screening of targets

The set of ATBC-interacting targets was compared with the set of liver damage-related targets, and Venn diagram analysis was used to identify the intersection of both and designate the overlap as a potential target specific to ATBC. Cytoscape software (v3.8.2) was utilized to construct a drug-disease common target network, with nodes representing targets and edges representing interactions between targets. Network topology analysis was then used to calculate the degree value of the nodes, median centrality and other parameters. This process enabled the identification of the key nodes (core targets) in the network.

#### 2.2.5 Gene function and pathway enrichment analysis of target protein

GO function enrichment analysis and KEGG pathway enrichment analysis of drug-disease common targets were performed using the DAVID database (Dennis et al., 2003) (https://david.ncifcrf.gov/). GO function enrichment analysis is a process of analysis of the biological functions involved in the common targets at three levels: biological process (BP), cellular composition (CC), and molecular function (MF) (Gene Ontology Consortium, 2015). KEGG pathway enrichment analysis is a process of identification of signaling pathways that are significantly enriched for common targets. A threshold of P < 0.05 was set for enrichment analysis to screen for statistically significant enrichment results.

#### 2.2.6 Molecular docking for ATBC and core targets

The Protein Data Bank (PDB) database (http://www.rcsb.org/) was utilized to obtain the three-dimensional structure of the key target, remove water molecules and other ligands, and hydrotreat. The structure of ATBC was drawn by ChemDraw software. Docking calculations were performed using AutoDock Vina software. We chose the default docking parameters, defined the binding site, and then docked the ATBC ligand to the target protein for simulation. Finally, Discovery Studio and Pymol were used to visualize the results.

#### 2.2.7 CCK-8 assay

Liver cells were inoculated into 96-well plates and cultured overnight. Different concentrations of ATBC solution (0, 10, 50, 100, 200, 500, 1,000 or 5,000 μM) were added to the experimental group, and an equal amount of medium was added to the control group. The incubation was continued for 36 h. 10 μL of CCK-8 reagent was added to each well, and the incubation was continued for 2 h. The absorbances were measured at 450 nm with an enzyme marker and the growth curves of the cells were plotted.

#### 2.2.8 RNA isolation and quantitative real-time PCR analysis

Liver cells were treated with DMSO control or ATBC for 36 h. Total RNA was extracted by Trizol (Invitrogen, Carlsbad, United States) reagent, and then the RNA concentration was determined by NanoDrop spectrophotometer. According to the manufacturer’s instructions, RNA was first reverse transcribed into cDNA (Lianchuan Bio) and then subjected to RT-qPCR using SYBR GreenER™qPCR SuperMix Universal (Invitrogen). GAPDH was used as a housekeeping control gene. The relative expression of different genes was calculated using the 2^−ΔΔCT^ method. The primer sequences are shown in Table 1.

**TABLE 1:** mRNA-specific primers of genes.

Gene	Primer	Sequence (5′-3′)
GAPDH	FORWARD	GGC​AAA​TTC​AAC​GGC​ACA​GTC​AAG
	REVERSE	TCG​CTC​CTG​GAA​GAT​GGT​GAT​GG
TNF-α	FORWARD	CAA​TGG​CGT​GGA​GCT​GAG​AGA​TAA​C
	REVERSE	TCT​GGT​AGG​AGA​CGG​CGA​TGC
Bax	FORWARD	GAT​GCG​TCC​ACC​AAG​AAG​CTG​AG
	REVERSE	CAC​GGC​GGC​AAT​CAT​CCT​CTG
Bcl2	FORWARD	TAC​GAG​TGG​GAT​GCG​GGA​GAT​G
	REVERSE	CCG​GGC​TGG​GAG​GAG​AAG​ATG

#### 2.2.9 Western blot

We isolated proteins from liver cells and determined their concentration using a bicinchoninic acid (BCA) protein assay kit according to the manufacturer’s instructions. Subsequently, the proteins were separated by sodium dodecyl sulfate polyacrylamide gel electrophoresis (SDS-PAGE) and transferred to PVDF membranes. The membranes were incubated with primary antibodies anti-bax (proteintech), anti-bcl2 (proteintech) and anti-β-actin (Santa Cruz Biotechnology), respectively, at 4°C overnight, and then incubated with the appropriate secondary antibodies for 1 h at room temperature. Enhanced chemiluminescence detection reagent was achieved for further visualization.

#### 2.2.10 Immunofluorescence

Liver cells were inoculated into Petri dishes placed with cell crawls and cultured overnight. The experimental group was treated with ATBC, while the control group was incubated with basal medium. At the end of the treatment, the cell crawls were removed and fixed with 4% paraformaldehyde for 30 min. Thereafter, the samples were washed three times by TBST and closed with goat serum protected from light at room temperature for 1 h. The samples were then incubated sequentially, with the addition of primary antibody, fluorescent secondary antibody, and finally sealed with solid medium containing DAPI. The samples were then imaged using confocal microscopy.

#### 2.2.11 Statistical analysis

Statistical analysis was conducted using GraphPad Prism version 9.0 software (GraphPad Software, Inc., La Jolla, CA, United States). All data were presented as the mean ± standard deviation. A two-tailed t-test was employed for comparative analyses between two groups. For multiple comparisons, analysis of variance (ANOVA) with Tukey’s correction was applied. P < 0.05 was considered statistically significant.

## 3 Results

### 3.1 Initial network assessment of ATBC toxicity

The toxicity model suggests that the active toxicity end point is related to oncogenicity, and the membrane transporter associated with ATBC is the blood-brain barrier (BBB). These findings are consistent with the real reports of ATBC mediated toxicity in humans in the previous literature, which lays the foundation for us to further study the toxic effects of ATBC on humans.

### 3.2 Screening of the underlying targets of ATBC and liver damage

**FIGURE 1:**
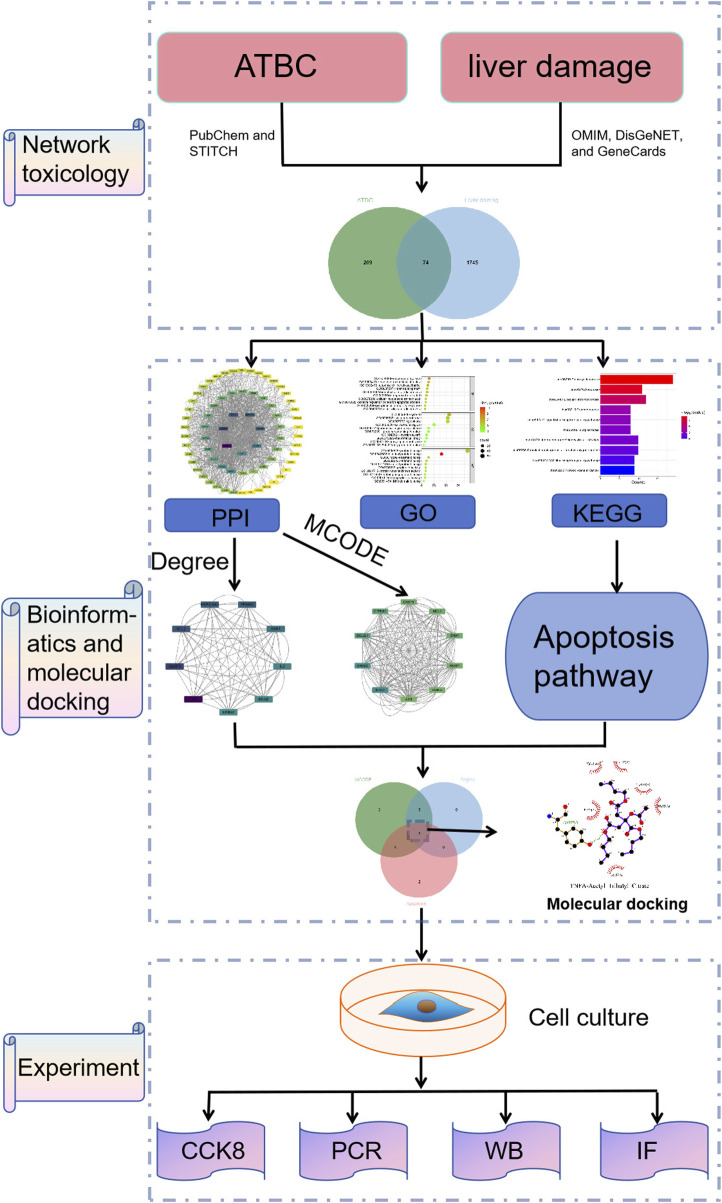
Flowchart of this study.

**FIGURE 2:**
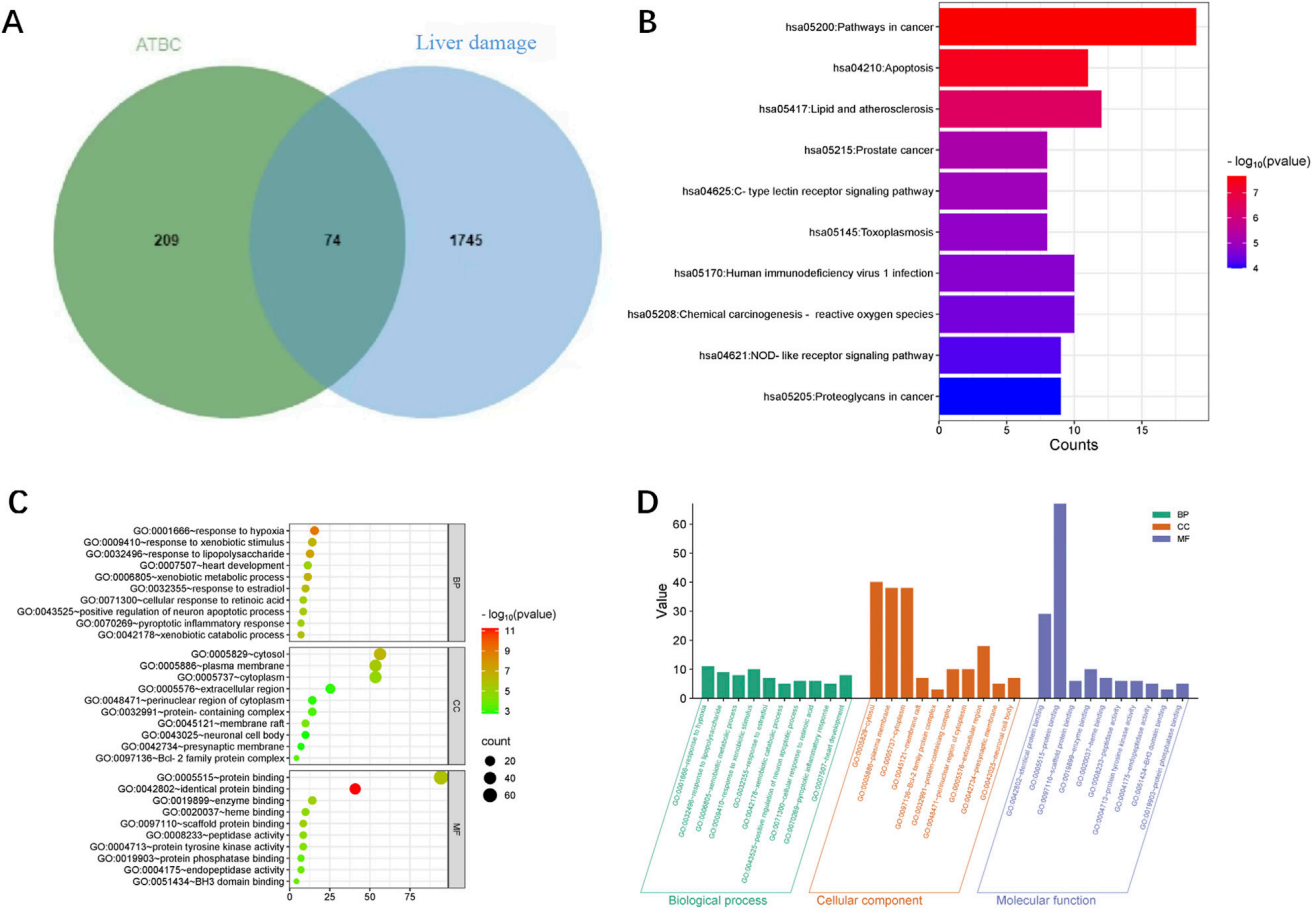
**(A)** Venn diagram of the targets of ATBC and liver damage. **(B)** KEGG enrichment analysis of potential targets (top 10). The histogram illustrated the frequency and signiffcance of enrichment for each pathway. The length of each bar corresponded to the gene counts, indicating enrichment score, with taller bars representing larger counts. The enrichment signiffcance was shown by the color saturation of the bar. **(C)** GO enrichment analysis of potential targets (top 10). The size of each bubble corresponded gene expressions in a particular pathway. The enrichment signiffcance was shown by the color saturation of the bubble. **(D)** This histogram illustrated the top 10 enriched entries for each GO category (BP, CC, and MF) with smaller P values on the 164 potential targets. The P values reffected the statistical signiffcance of the enrichment, with smaller values indicating higher signiffcance. The height of each bar corresponds to the P values, reffecting the degree of enrichment within the respective category. These enriched entries highlighted keybiological processes, cellular components, and molecular functions that are potentially inffuenced by ATBC exposure.

The experimental design of the present study is shown in Figure 1. A total of 287 action targets of ATBC were identified through searches of the CHEMBL and STITCH databases. After de-duplication and screening, 283 human gene targets were retained. Additionally, 1819 liver damage-related targets were sourced from the OMIM and GeneCards databases. A comparative analysis of these two sets revealed 74 common targets associated with both ATBC and liver damage, as illustrated in Figure 2A.

### 3.3 GO function and KEGG pathway analyses

To explore the mechanism of toxicity, DAVID database was employed to perform enrichment analysis of the 74 targets. The results of GO functional enrichment analysis indicated that, concerning biological processes, the common targets were primarily enriched in metabolic processes, oxidative stress responses, regulation of inflammatory responses, and apoptotic cell death; Regarding cellular composition, these targets were predominantly associated with structures such as the cytoplasm and cell membrane. In terms of molecular function, the targets were primarily implicated in protein kinase activity, ferrous heme binding, as well as the binding of proteins, enzymes, and other substances (Figures 2C,D; Table 2). These findings suggest that ATBC may influence liver cell structure and function by affecting the aforementioned biological processes and molecular functions, potentially leading to liver damage. KEGG pathway enrichment analysis revealed that the common targets were significantly enriched in multiple signaling pathways, with the top five pathways being the cancer pathway, apoptosis signaling pathway, lipid metabolism and atherosclerosis, prostate cancer, and C-type lectin receptor signaling pathway (Figure 2B; Table 3). Remarkably, the apoptosis signaling pathway was among the most enriched, indicating that ATBC may contribute to the development of liver damage by modulating apoptosis.

**TABLE 2:** Specific p-values or false discovery rates (FDR) in GO analysis.

Term	Category	P value	FDR
GO:0001666∼response to hypoxia	BP	6.934159078461771E-10	1.02E-06
GO:0032496∼response to lipopolysaccharide	BP	4.535742978836638E-8	3.34E-05
GO:0006805∼xenobiotic metabolic process	BP	2.142580563118749E-7	1.05E-04
GO:0009410∼response to xenobiotic stimulus	BP	3.075141307797081E-7	1.13E-04
GO:0032355∼response to estradiol	BP	6.49791734901172E-7	1.91E-04
GO:0042178∼xenobiotic catabolic process	BP	1.228968263744642E-6	7.77E-04
GO:0043525∼positive regulation of neuron apoptotic process	BP	3.695663519101649E-6	7.77E-04
GO:0071300∼cellular response to retinoic acid	BP	5.310694499198932E-6	9.04E-04
GO:0070269∼pyroptotic inflammatory response	BP	5.529140390443222E-6	9.04E-04
GO:0007507∼heart development	BP	9.577082584908523E-6	0.001408789
GO:0005829∼cytosol	CC	3.5764096589106503E-7	6.58E-05
GO:0005886∼plasma membrane	CC	3.20378382203122E-6	2.95E-04
GO:0005737∼cytoplasm	CC	1.5166837324232216E-5	9.30E-04
GO:0045121∼membrane raft	CC	8.678122131636208E-5	0.003991936
GO:0097136∼Bcl-2 family protein complex	CC	4.009459929943385E-4	0.014754813
GO:0032991∼protein-containing complex	CC	5.269870616265552E-4	0.016160937
GO:0048471∼perinuclear region of cytoplasm	CC	9.975395230141537E-4	0.025384763
GO:0005576∼extracellular region	CC	0.001103685326918287	0.025384763
GO:0042734∼presynaptic membrane	CC	0.001605120600771092	0.031560491
GO:0043025∼neuronal cell body	CC	0.0017152440506773266	0.031560491
GO:0042802∼identical protein binding	MF	5.4654933140631765E-12	2.12E-09
GO:0005515∼protein binding	MF	1.2721255886048492E-6	2.47E-04
GO:0097110∼scaffold protein binding	MF	6.436720615904623E-6	8.09E-04
GO:0019899∼enzyme binding	MF	8.337673951813218E-6	8.09E-04
GO:0020037∼heme binding	MF	2.225584023547962E-5	0.001699569
GO:0008233∼peptidase activity	MF	2.6281994273140062E-5	0.001699569
GO:0004713∼protein tyrosine kinase activity	MF	5.7534676132851376E-5	0.003189065
GO:0004175∼endopeptidase activity	MF	1.6173309394584072E-4	0.007844055
GO:0051434∼BH3 domain binding	MF	1.9453289700304796E-4	0.008386529
GO:0019903∼protein phosphatase binding	MF	3.1267884056038615E-4	0.012131939

**TABLE 3:** Specific p-values or false discovery rates (FDR) in KEGG analysis.

Term	P value	FDR
hsa05200:Pathways in cancer	2.1349730148751515E-8	2.5794872541278018E-6
hsa04210:Apoptosis	3.878928201695943E-8	2.5794872541278018E-6
hsa05417:Lipid and atherosclerosis	3.3349230060716643E-7	1.4784825326917712E-5
hsa05215:Prostate cancer	6.035207994392361E-6	2.00670665813546E-4
hsa04625:C-type lectin receptor signaling pathway	9.544308451276436E-6	2.538786048039532E-4
hsa05145:Toxoplasmosis	1.4602545174876136E-5	3.2368975137642104E-4
hsa05170:Human immunodeficiency virus 1 infection	1.9722294128746236E-5	3.747235884461785E-4
hsa05208:Chemical carcinogenesis - reactive oxygen species	3.266340204395599E-5	5.430290589807684E-4
hsa04621:NOD-like receptor signaling pathway	5.8667818154328844E-5	8.669799793917485E-4
hsa05205:Proteoglycans in cancer	1.0039955480317328E-4	0.0013353140788822046

### 3.4 Construction and analysis of protein interaction networks

**FIGURE 3:**
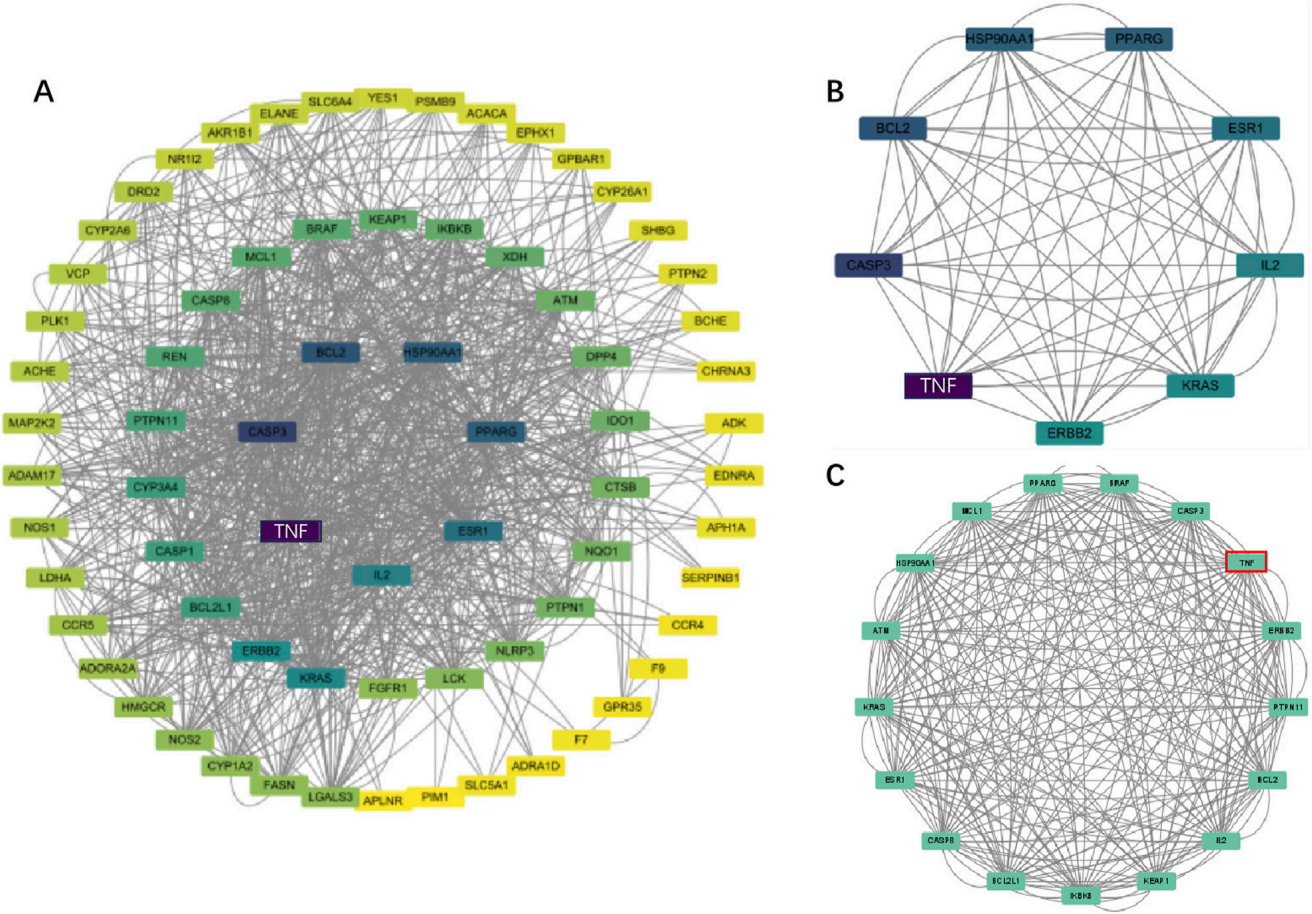
**(A)** The PPI network of potential targets. **(B)** Top 9 genes with the highest degree values were found using CytoHubba_Degree and the depth of the color correspond to the weighted score. **(C)** The densest connected region in the PPI network was identiffed using MCODE.

**FIGURE 4:**
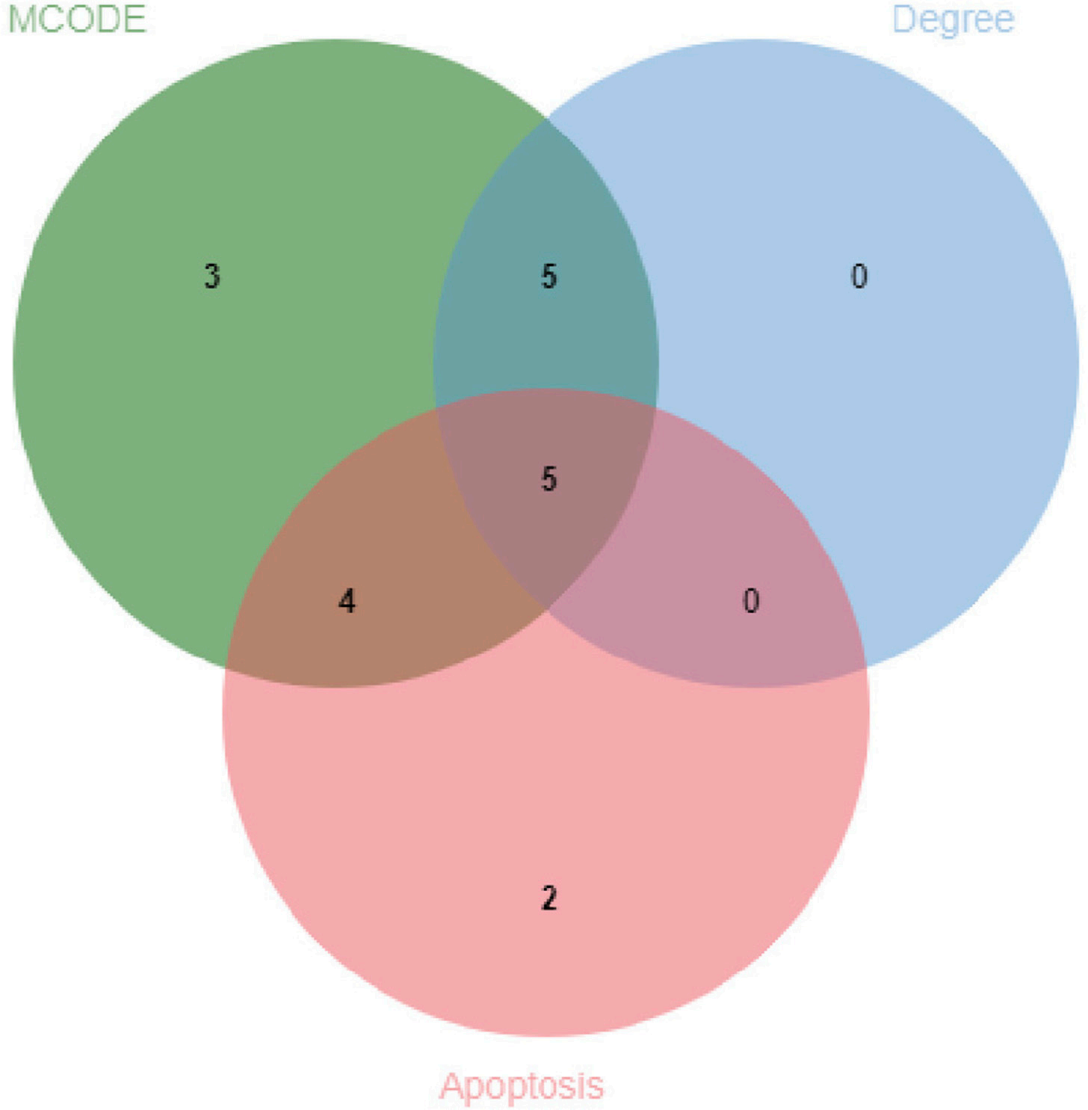
Venn diagram of CytoHubba_Degree, MCODE and apoptotic molecules.

The protein-protein interaction (PPI) network constructed using Cytoscape software comprised 71 nodes and 934 edges (Figure 3A). Through network topology analysis, the top nine core targets were identified based on their degree values: TNF, CASP3, BCL2, HSP90AA1, PPARG, ESR1, IL2, KRAS, and ERBB2, in that order (Figure 3B; Table 4). These core targets occupy crucial positions within the network and exhibit extensive interactions with other targets, suggesting their significant roles in the process of ATBC-induced liver damage. Additionally, MCODE plug-in demonstrated the hub module of these nodes, consisting of BCL2L1, CASP8, ESR1, KRAS, ATM, HSP90AA1, MCL1, PPARG, BRAF, CASP3, TNF, ERBB2, PTPN11, BCL2, IL2, KEAP1, IKBKB (Figure 3C). KEGG analysis has demonstrated that apoptosis probably plays a vital role in ATBC-mediated hepatotoxicity. Thus, we intersected the genes enriched in apoptosis term in KEGG analysis with the targets from the MCODE and Degree algorithms. The results confirmed that there were five targets involved ATBC-induced hepatocyte apoptosis, including BCL2L1, KRAS, CASP3, TNF, BCL2 (Figure 4). Notably, TNF was identified as the top target with the highest degree value. In summary, ATBC may exert its’ hepatotoxicity via induing hepatocyte apoptosis via targeting TNF-α.

**TABLE 4:** The top nine targets ranked by Degree score.

Targets name	Degree score
TNF	100
CASP3	78
BCL2	72
HSP90AA1	68
PPARG	68
ESR1	62
IL2	56
KRAS	54
ERBB2	52

### 3.5 Molecular docking for ATBC and TNF-α protein of liver damage

**FIGURE 5:**
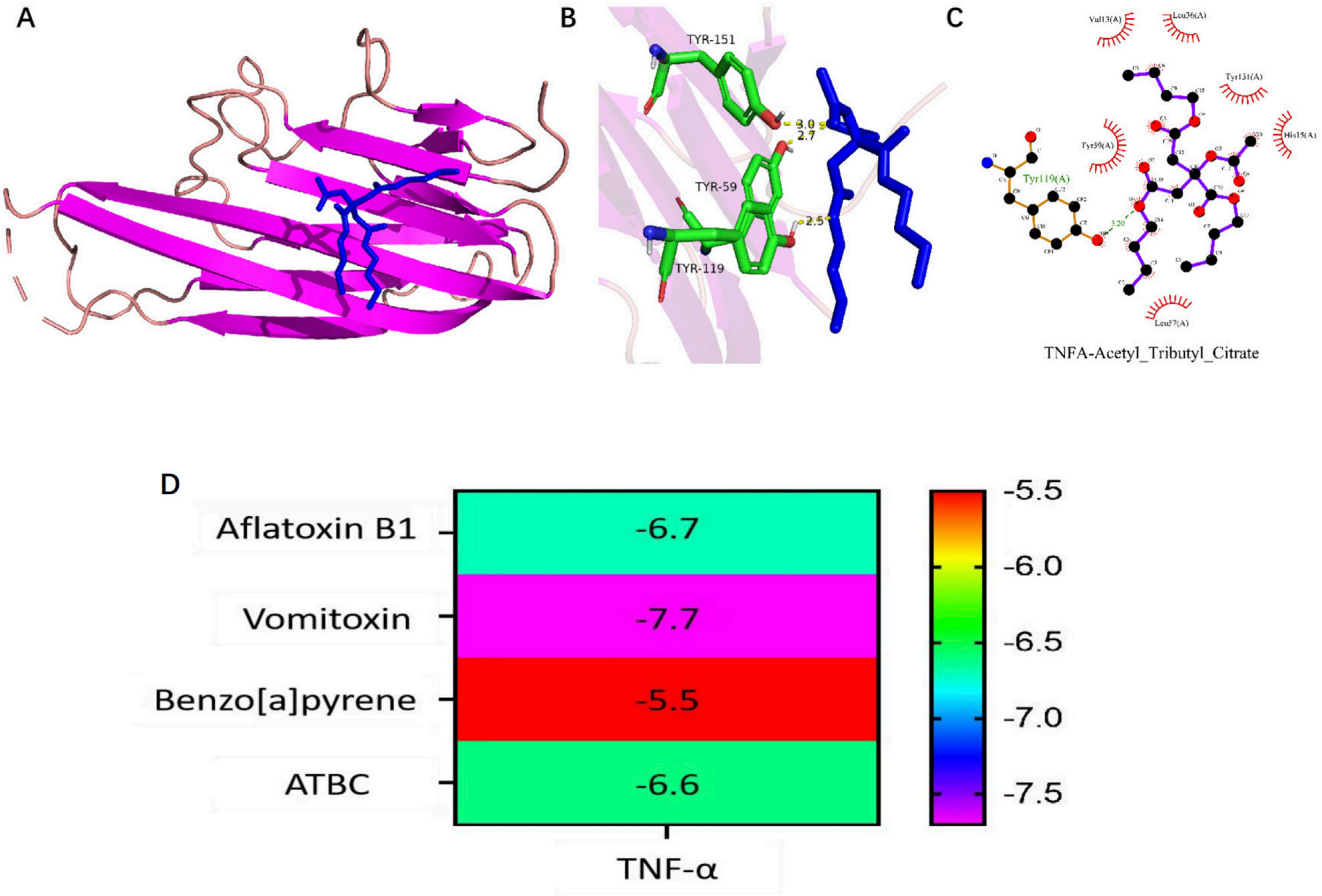
**(A–C)** Molecular docking results of TNF-α with the Acetyl tributyl citrate (ATBC). **(D)** Heatmap of molecular docking binding energy of ATBC to core target TNF-α.

To investigate the binding activity between ATBC and the key target, as well as to confirm potential interaction, molecular docking experiment was conducted for TNF-α against ATBC. The AutoDock software was employed to generate docking results exhibiting favorable binding energies, demonstrating that ATBC forms stable binding conformations with TNF-α (Affinity ability: −6.6 kcal/mol) (Figure 5). This suggests a strong affinity of ATBC for TNF-α, corroborating the results of network toxicology predictions at the molecular level.

### 3.6 ATBC affected cell proliferation

**FIGURE 6:**
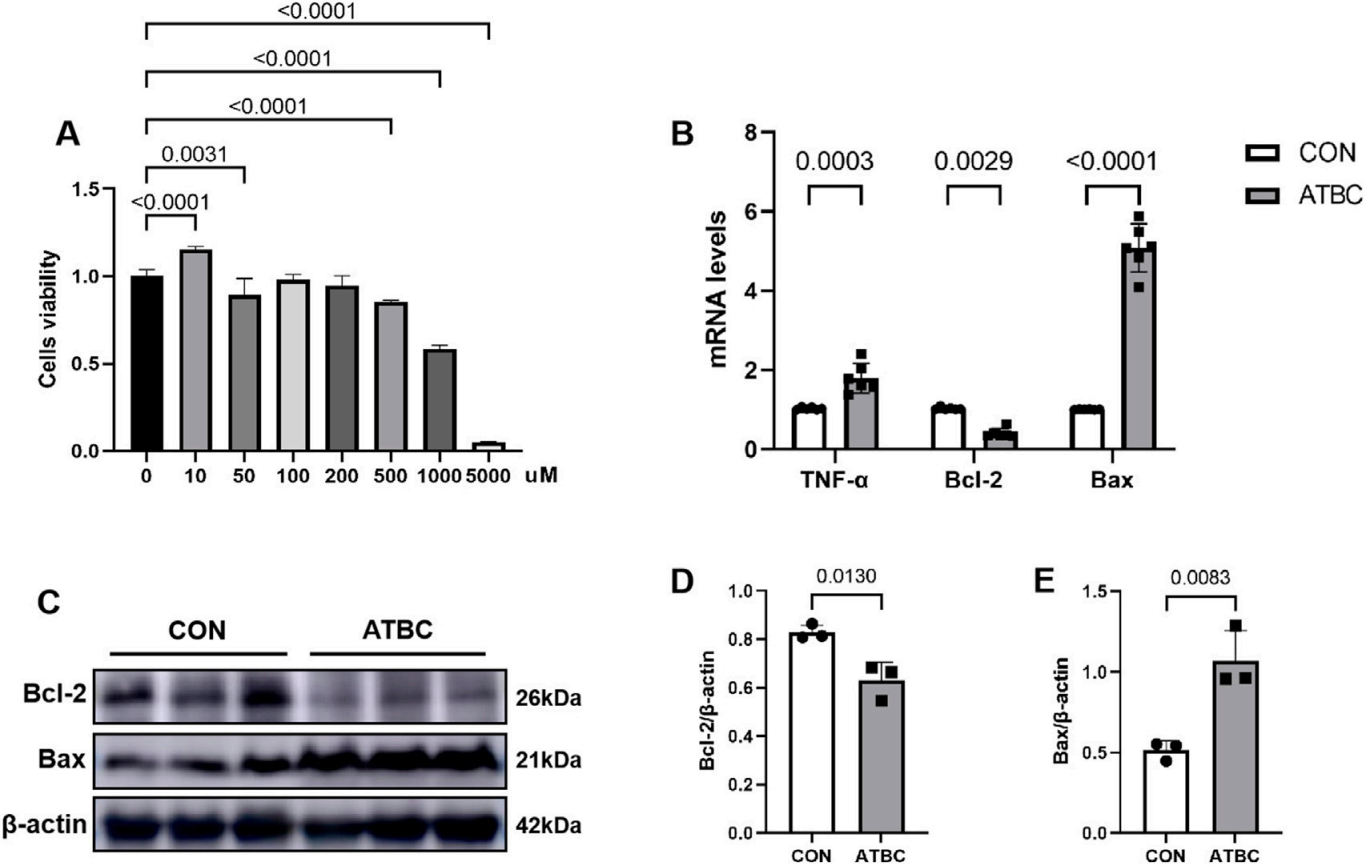
ATBC affected cell proliferation. **(A)** CCK-8 assay was used to determine the optimal concentration of ATBC for hepatotoxicity. **(B)** The mRNA expression levels of TNF-α, Bcl2, and BAX. **(C–E)** The protein expression levels of Bcl2 and BAX.

To evaluate the effect of ATBC on liver cell viability and activity, an *in vitro* model was established using mouse liver cells. The proliferative activity of these cells was assessed using the CCK-8 assay. The cells were exposed to various concentrations of ATBC, ranging from 0 to 5,000 μM, for 24 h. The results indicated that a concentration of 10 μM of ATBC promoted cellular growth, while higher concentrations exhibited an inhibitory effect on proliferation (Figure 6A). Notably, a concentration of 1,000 μM of ATBC had the observably inhibitory effect on cell viability, which was subsequently selected for further experiments.

### 3.7 ATBC affected the expression of the core proteins and genes

**FIGURE 7:**
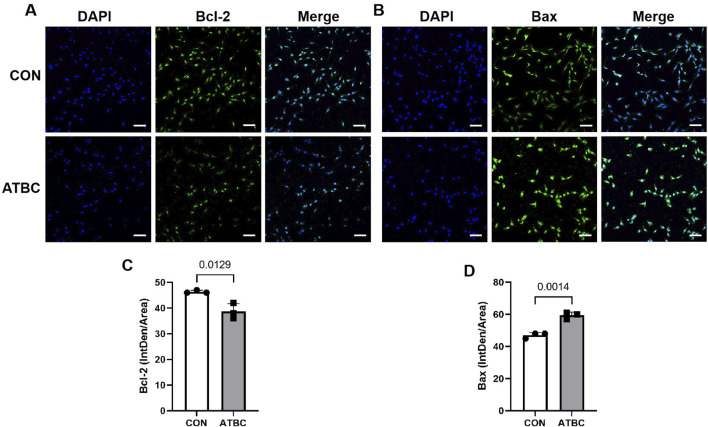
Expression and localization of target proteins. Immunofluorescence showed that the expression of Bcl2 **(A,C)** was significantly increased in ATBC group, while the expression of BAX **(B,D)** was significantly reduced in ATBC group.

To verify changes in the expression of key gene and apoptosis-related markers identified in the network analysis in response to ATBC-induced liver damage, qRT-PCR experiments were conducted. These revealed that the mRNA expression levels of Bax and TNF were significantly up-regulated compared to the control group (P < 0.05), while the expression levels of Bcl2 was significantly reduced (Figure 6B), consistent with their predicted involvement in network toxicology related to liver damage processes and signaling pathways. Furthermore, Western blot (WB) experiments demonstrated that the expression patterns of these proteins aligned with the qRT-PCR results. Specifically, the level of Bax protein was significantly elevated, whereas the level of Bcl2 protein was significantly diminished following ATBC treatment (Figures 6C–E). Additionally, cellular immunofluorescence revealed enhanced fluorescence intensity of apoptosis markers (Bax) proteins with altered intracellular distribution in the ATBC-treated group, while the fluorescence intensity of Bcl2 was significantly reduced (Figures 7A–D). This provides compelling visual evidence for the impact of ATBC on the expression and intracellular localization of these apoptosis related proteins, thus offering cellular-level support for the findings of the cytotoxicity analysis.

## 4 Discussion

ATBC, a widespread plasticizer, is used to enhance the flexibility and processability of plastic materials (Chung et al., 2019). Despite its functional benefits in materials science, the lipophilicity and environmental persistence of ATBC raise concerns regarding its accumulation and potential endocrine-disrupting effects in biological systems (Martínez-Ibarra et al., 2021). This study systematically screened 74 potential targets associated with ATBC-induced liver damage using various databases. Utilizing the STRING platform and Cytoscape, an interaction network was constructed, and five key nodes (BCL2L1, KRAS, CASP3, TNF, and BCL2) were identified. The mutual binding of these nodes was confirmed through molecular docking experiments. Furthermore, the potential health implications of environmentally relevant levels of ATBC were investigated using *in vitro* exposure models. The exposure experiments demonstrated that ATBC concentrations of 50, 100, 200, 500, 1,000, and 5,000 µM led to a significant reduction in the viability of mouse liver cells. Additionally, the expression levels of key molecules were found to be consistent with cyber-toxicological predictions through PCR, WB, and immunofluorescence techniques.

Apoptosis, a form of programmed cell death, is essential for maintaining cellular homeostasis and ensuring normal liver function (Kerr et al., 1972). The apoptosis process can be categorized into two distinct pathways: the exogenous apoptotic pathway and the endogenous apoptotic pathway. The exogenous pathway, or exogenous apoptosis, is defined as a signaling pathway initiated by the binding of transmembrane receptors, known as death receptors, to their ligands. TNF-α induces apoptosis via this pathway (Vanden et al., 2015), while the endogenous pathway is tightly regulated by the Bcl-2 protein family, which operates at upstream and mitochondrial levels to integrate death and survival signals (Cor et al., 2002; Youle and Strasser, 2008). In the early phase, following the binding of TNF-α to TNFR1, both TNFR1-associated TRADD and TNFR1-associated RIPK1 recruit TNF receptor-associated factor 2 (TRAF2) and the TRAF2- interacting E3 ligases cellular inhibitor of apoptosis 1 (cIAP1) and cIAP2, resulting in K63-ubiquitination of TNFR1 signaling complex components (Lafont et al., 2018; Kupka et al., 2016). The linear multimeric ubiquitin chain-recruiting IKK complex is formed through the linear ubiquitin chain assembly complex (LUBAC). Upon activation of IKK, phosphorylated cytoplasmic IκBα undergoes ubiquitination modification, which is subsequently degraded by the proteasome and results in the nuclear translocation of NF-κB transcription factors and transcription of NF-κB-regulated genes (Zhang et al., 2017), which induces the release of IL-6 and CXCL8, among other chemokines, and the expression of the anti-apoptotic protein c-IAP1/2. In this study, targets linked to ATBC-induced liver damage were found to be significantly enriched in apoptosis-related pathways, with CASP3, a core gene for executing apoptosis, occupying a key position within the target network. This suggests that ATBC may induce hepatocyte programmed death by activating various apoptotic signaling pathways.

Current research on ATBC-induced hepatotoxicity is limited, however, the mechanism can be speculated in connection with similar plasticizers, such as Di (2-ethylhexyl) phthalate (DEHP). Multiple lines of evidence suggest that DEHP mediates hepatocyte apoptosis through the mitochondrial intrinsic pathway (Rowdh et al., 2018; Ha et al., 2016). For instance, Jiao et al. showed that exposure to elevated concentrations of monobutyl phthalate may lead to excessive production of reactive oxygen species (ROS) within the hepatic oxidative system, resulting in cellular toxicity and decreased cell viability (Jiao et al., 2020). Thus, we can hypothesize that ATBC may induce the release of cytochrome C from the mitochondrial matrix into the cytoplasm by disrupting the mitochondrial membrane potential—either by downregulating the membrane localization of the anti-apoptotic protein Bcl-2 or by promoting the oligomerization of the pro-apoptotic protein Bax/Bak. It has been established that the substance binds to Apoptotic Protease Activating Factor 1 (APAF-1) to form apoptotic bodies (Orrenius et al., 2015), leading to the recruitment and activation of Procaspase-9, which activates the caspase cascade. This triggers CASP3 activation, culminating in typical apoptotic features such as nuclear condensation and DNA fragmentation.

The inflammatory response is a significant pathological feature of liver damage. In this study, TNF was identified as one of the nodes most closely linked to ATBC-induced hepatotoxicity. As core mediators of the inflammatory response, TNF molecules play dual roles in ATBC-mediated liver damage: ATBC promotes the release of pro-inflammatory factors, such as TNF-α, by activating immune cells (e.g., Kupffer cells) or directly affecting liver cells. Pro-inflammatory factors further stimulate inflammatory cells, exacerbating the inflammatory response and creating a vicious cycle (Lacey et al., 2018). This ultimately leads to immune cell infiltration and hepatocyte dysfunction in liver tissues.

Notably, the TNF signaling pathway has been shown to induce apoptosis in liver cells through extrinsic apoptotic pathways. TNF-α can also cause caspase-independent necrotic cell death (necrotic prolapse), a process involving ROS production from mitochondrial or non-mitochondrial sources (Vandenabeele et al., 2010). Upon activation by TNF-R1, caspase-8 binds to ROMO-1, a regulator of ROS located in the outer mitochondrial membrane (OMM) (Blaser et al., 2016). This binding causes ROMO1 to sequester Bcl-XL, triggering a loss of mitochondrial membrane potential and increasing ROS production. Furthermore, it has been demonstrated that mitochondrial ROS production can be triggered by a complex JNK-mediated mechanism (Blaser et al., 2016), but its activity is inhibited by TRAF2 and cIAPs, as well as by kinases of the NF-κB signaling pathway, such as IKK2 and TAK1, and genes transcriptionally up-regulated by this pathway, such as genes encoding cell survival proteins, including caspase 8 and FADD-like apoptosis regulators, the anti-apoptotic BCL2 family members, such as BFL1 and BCL-XL, as well as cIAP2, A20 and H-ferritin (Wajant et al., 2003). Exposure to DEHP has been reported to lead to MEHP accumulation in the liver, activation of TNF/TNFR1 pathway-mediated cellular pyroptosis, and upregulation of the pore-forming protein Gasdermin D (GSDMD-N). This disrupts the mitochondrial membrane of liver cells, resulting in cell death (Zhang et al., 2025). It is evident that TNF signaling exhibits a switchable property between pro-inflammatory and pro-apoptotic functions, with this functional switch potentially closely related to the dose and duration of ATBC exposure.

At present, six biologic drugs that inhibit the TNF-TNFR1-TNFR2 system are approved for clinical use, and infliximab (Ib) is one of them, whose primary mode of action is to neutralise TNF. A previous study reported the protective effect of Ib against paracetamol-related hepatotoxicity, suggesting that Ib administration significantly reduced serum alanine aminotransferase (ALT), aspartate aminotransferase (AST), and TNF-α levels (Ferah et al., 2013). However, there is an absence of clinical or basic studies demonstrating that Ib attenuates ATBC-induced liver injury. It has been hypothesised that Ib may attenuate cellular injury by decreasing ROS and cytokine levels (Cure et al., 2015; Amanzada et al., 2014) or ameliorate CTC-induced hepatic injury by decreasing the production of transforming growth factor β and interleukin 1β (IL-1β) and modulating purine metabolism (Sehitoglu et al., 2015). Consequently, the present study hypothesises that the targeting of TNF to ameliorate ATBC-associated hepatotoxicity may be related to anti-inflammatory antioxidants.

In this study, we aimed to confirm the hypothesis that ATBC and TNF-α exhibit strong affinity through molecular docking experiments. Additionally, PCR experiments were employed to detect significantly elevated TNF gene expression following ATBC treatment. This finding further substantiates ATBC-mediated inflammatory responses leading to liver damage. The PCR, WB, and fluorescence experiments confirmed the involvement of the apoptotic molecule Bax and the anti-apoptotic molecule Bcl2 in ATBC-induced hepatocyte death, at both the mRNA and protein levels.

The primary strength of this study lies in its use of network toxicology as a nascent technology and the incorporation of multiple databases to systematically analyze the potential mechanisms of ATBC-induced liver damage. This analysis reveals the intricate relationship between ATBC and liver damage-related targets and signaling pathways, providing a comprehensive theoretical framework for future research. Furthermore, molecular docking was used to validate the binding activity of ATBC with pivotal targets at the molecular level. A diverse array of experiments, including PCR, WB, and cellular immunofluorescence, was conducted to corroborate the findings of the network analysis across gene, protein, and cellular levels. This multifaceted approach enhances the credibility and persuasiveness of the research conclusions. Nevertheless, it is essential to acknowledge the limitations of this study. The network toxicology analysis relies on bioinformatics-based predictions, and while experimental validations followed, these may not fully reflect real biological processes. In addition, Surface plasmon resonance (SPR) technology could offer a more robust method of determining the binding of ATBCs to their targets. In the experimental phase, only cells were selected for study, omitting *in vivo* animal experiments to further validate the damaging effects and mechanisms of ATBC on the liver. Differences between cell experiments and the *in vivo* conditions may lead to biased results. Moreover, this study only validated a subset of the key targets and signaling pathways, leaving some predicted results unexamined. Therefore, further studies are recommended to broaden the scope of validation and explore the specific mechanisms underlying ATBC-induced liver damage.

## 5 Conclusion

In summary, this study combined network toxicology and molecular docking techniques to investigate the molecular mechanisms and pathways of ATBC toxicity to the liver comprehensively. Through PCR, WB, and immunofluorescence experiments, we demonstrated that ATBC exerts a deleterious effect on the liver. Furthermore, we established that ATBC influences the expression of relevant genes and proteins within the liver, instigates inflammatory responses, precipitates the apoptosis of hepatocytes, and disrupts normal liver physiological functions. This multi-faceted approach provides comprehensive evidence of the mechanisms underlying ATBC-induced liver damage and lays a solid theoretical foundation for assessing the health risks associated with ATBC and preventing liver diseases.

## Data Availability

The datasets presented in this study can be found in online repositories. The names of the repository/repositories and accession number(s) can be found in the article/supplementary material.
